# Implementing the European Core Health Indicators (ECHI) in the Netherlands: an overview of data availability

**DOI:** 10.1186/s13690-014-0058-4

**Published:** 2015-03-02

**Authors:** Maartje Marloes Harbers, Marieke Verschuuren, Agnes de Bruin

**Affiliations:** RIVM, National Institute for Public Health and the Environment, P.O. Box 1, 3720 BA Bilthoven, the Netherlands; CBS, Statistics Netherlands, P.O. Box 24500, 2490 HA Den Haag, the Netherlands

**Keywords:** Public health, Monitoring, European Core Health Indicators, International comparisons, European Union, Netherlands

## Abstract

**Background:**

The European Commission, together with the European Union (EU) Member States, developed a core set of indicators for monitoring public health in the EU, the European Core Health Indicators (ECHI) shortlist. From 2009 to 2012 developmental work on the ECHI indicators continued within the framework of the Joint Action for European Community Health Indicators and Monitoring (ECHIM). In this article, we give the current state of affairs on the availability of data for the ECHI indicators in the Netherlands and show what progress has been made over the past 5 years. The information provided serves as an illustration of the challenges encountered in a European country when working on harmonising national data collections with international data delivery requirements.

**Methods:**

To assess data availability, we consulted Dutch data experts and relevant websites and reports on health monitoring activities. We compared the available Dutch data with the definitions, preferred data sources and relevant dimensions as requested by ECHI.

**Results:**

The Netherlands can provide data for 66 of the 75 ECHI indicators for which availability could be assessed: for all of the 48 ECHI indicators that can be extracted from international databases and for 18 of the 27 indicators not available from international databases. Breakdowns by socio-economic status and region are not possible for 23 (35%) of the total of 66 indicators for which data are available and for 21 (32%) of these 66 indicators the definition is not exactly the same as requested by ECHIM. Since 2009, better estimates have become available for low birth weight, practising physicians and practising nurses.

Moreover, several European initiatives to improve harmonised data collection are expected to have a positive effect on data availability for the Netherlands. Such initiatives should become sustainable in order to provide possibilities for monitoring trends. The scattered data ownership in the Netherlands complicates the coordination work for international data deliveries.

**Conclusion:**

Data availability in the Netherlands is good. Since 2009, several Dutch and European developments in harmonising data collection have contributed or will significantly contribute to improvements in the data situation for the ECHI indicators in the Netherlands.

**Electronic supplementary material:**

The online version of this article (doi:10.1186/s13690-014-0058-4) contains supplementary material, which is available to authorized users.

## Background

Public health indicators are an indispensable tool for providing an evidence base for the development and evaluation of public health policies. Moreover, monitoring public health by means of a clear set of core indicators also contributes to transparency and good governance. Additionally, by making sure that indicators are comparable across countries, possibilities for benchmarking and identifying good practice examples or problems arise.

For these reasons, the European Commission, together with the European Union (EU) Member States, started in the late nineties with the development of a core set of indicators for monitoring public health in the EU. The first version of this core set was published in 2005 and was named the ECHI (European Community Health Indicators) shortlist [1]. The ECHI shortlist was updated in 2008 [2] and in 2012. The current, 2012 version, consists of 88 indicators in different states of development [3]. In 2013 the ECHI indicators were renamed European Core Health Indicators and an evaluation of the use and impact of ECHI concluded that there is considerable consensus among stakeholders on establishing a permanent health indicator system like ECHI at the European level, possibly with the joint involvement of the Organisation for Economic Co-operation and Development (OECD), the World Health Organization (WHO) and Eurostat [4]. In addition the Council of the European Union in its Council conclusions of 10 December 2013 welcomes further development and consolidation of a health monitoring and information system at EU level based on the European Core Health Indicators and possibly within the scope of a European health information research infrastructure consortium [5]. A logical next step would be to integrate the ECHI indicators into the common European Health Information System that is currently being developed by the European Commission, WHO and Eurostat [6].

The shortlist as a whole covers the public health field in a comprehensive way, i.e. the shortlist contains indicators on health status, health determinants, health systems/health services and demographic and background variables. Selection criteria underlying the ECHI shortlist were related to policy relevance and potential policy impact, at both EU and Member State level, and to the magnitude of the public health problems considered. As much as possible, indicators from existing indicator sets were used (e.g. Eurostat, the WHO Health for All database (WHO-HfA) and the OECD health indicators). Nevertheless, the set was not data-driven and there was room for innovations. Such innovations could be based both on new scientific insights and new policy needs [7].

As of 2005, EU Member States as well as the European Commission started with the implementation of the ECHI shortlist, i.e. putting the indicators into practical use. Two main components can be discerned in national implementation activities: 1) mapping and improving the availability of national data for the ECHI indicators, and 2) actually using the indicators in national monitoring and reporting activities. Improving data availability includes improving the alignment of national indicator definitions with the ECHI definitions and calculations, or improving possibilities for breaking down data according to the subgroups requested by ECHI [8].

In the Netherlands, a start was made with the implementation of the ECHI shortlist by drafting the Dare to Compare! report, which was published in 2008 [9]. This report compares the health of the Dutch citizen with that of other Europeans by means of the ECHI indicators. Furthermore, the availability of data in the Netherlands was assessed. Since 2009, developmental work on the ECHI indicators continued within the framework of the Joint Action for European Community Health Indicators and Monitoring (ECHIM), resulting in more and better-refined indicator definitions [10]. Therefore, in 2011–2013, the ECHI-team of the Dutch National Institute for Public Health and the Environment (RIVM), with the help of Statistics Netherlands, updated the overview of availability of data for the ECHI indicators in the Netherlands. The aim of this updated overview was to answer the following questions:

1. What is the current state of affairs on the availability of Dutch data for the ECHI indicators?
2. What is the progress in improving the availability of Dutch data for the ECHI indicators over the past 5 years?

The outcomes of the updated availability overview for the Netherlands will be presented in this article. The overall results and conclusions of the Joint Action for ECHIM are described elsewhere [10].

Although the current article describes the data availability in the Netherlands, the information provided in this article can be seen as an illustration of the challenges encountered in an EU Member State when working on harmonising national data collections with international data delivery requirements. Especially within the EU this is a topic of importance, as data collections for indicator sets such as ECHI are becoming more and more obligatory under the legal framework provided by the 2008 Regulation on Community statistics on public health and health and safety at work [11]. In addition, several of the indicator developments that we describe (European Health Interview Survey (EHIS), European Health Examination Survey (EHES) and Eurostat morbidity statistics activities) not only have an impact on the Dutch data availability but are also relevant for other EU countries.

## Methods

For the overview of data availability, we compared the available Dutch data with the definitions, preferred data sources and relevant dimensions as described in the ECHI indicator documentation sheets [3]. We assessed both general data availability as well as the alignment of national indicator definitions with the ECHI definitions and calculations, and possibilities to break down by the subgroups requested by ECHI. The extent to which information on the alignment of national indicator definitions with the ECHI definitions and calculations is available, differs between indicators. Therefore, the level of detail with which disparities from the ECHI indicator definitions could be assessed also differs. Quality issues such as validity are not included in this assessment.

Information on available data was gathered through a desk research. Between 2011 and 2013 we consulted Dutch data experts and relevant websites and reports on national and international monitoring activities (e.g. http://statline.cbs.nl [12], www.nationaalkompas.nl [13] and the report “Netherlands Pilot Project on Morbidity Statistics” [14]). The results were cross-referenced by data experts from Statistics Netherlands. The overview focused on 67 of the 88 indicators for which ECHI indicator definitions are finalized and for which a decision was made on the preferred data source. For 8 of these 67 indicators there are two indicator operationalisations: one based on data from health interview surveys (HIS) and one based on national administrative or register data. In the remainder of this article we will count these two operationalisations as separate indicators, meaning that we describe 75 rather than 67 indicators (see Table 1 and Additional file 1). See Additional file 2 for the 21 indicators for which the indicator definition was lacking or for which no preferred data source was established yet at the start of the Joint Action in 2009.

**Table 1:** **Overview of data availability for 75 ECHI shortlist indicators in the Netherlands, 2011-2013**

**Availability**	**Indicators in international databases (N = 48)**	**Indicators not (yet) available in international databases (N = 27)**
Data available, definition (practically) the same as ECHI, possible to have all requested breakdowns/dimensions	27	4
Data available, definition (practically) the same as ECHI but some problems with requested breakdowns/dimensions	9	5
Data available, problems with definition but possible to have all requested breakdowns/dimensions	10	2
Data available, but problems with definition and with requested breakdowns/dimension	2	7
No data available	0	6
Uncertain: Data situation could not be assessed within the Joint Action for ECHIM	0	3

We distinguish between indicators for which data are already incorporated in regular international databases/data collections, and indicators for which data are not yet incorporated in international databases. For 48 of the 75 indicators data are incorporated in international databases such as the Eurostat database, the WHO-HfA database and the OECD Health database and data collections organised by the European Centre for Disease Prevention and Control (ECDC), the International Agency for Research on Cancer (IARC) or other international agencies or institutions. In this article, we will start with a description of the availability of Dutch data in the international databases for these 48 indicators.

For the remaining 27 of the 75 indicators no data were regularly collected at the international level at the start of the Joint Action for ECHIM. For these indicators we identified appropriate national Dutch data sources within the framework of the ECHIM Pilot Data Collection. The ECHIM Pilot Data Collection took place between July 2010 and April 2011 and focused for a large part on collecting national HIS data for countries like the Netherlands that did not (fully or not at all) implement the European Health Interview Survey (EHIS) in the period 2006–2009 (the first wave of EHIS). In addition to HIS data, within this pilot data were also collected for indicators for which national administrative or register-based sources are the preferred source. In this article, we will also describe the availability of the national HIS data and of the data from national administrative or register-based sources for the 27 indicators that are not regularly collected at the international level.

Finally, to assess the progress in improving the availability of Dutch data for the ECHI indicators, we identified relevant developments regarding the data availability in the Netherlands that have occurred since the publication of Dare to Compare! in 2008 [9].

## Results

### Dutch data available for 66 indicators

Table 1 presents an overview of the availability of data for the ECHI shortlist indicators in the Netherlands. For all of the 48 ECHI indicators that can be extracted from international databases, data for the Netherlands are available. The main data sources for these 48 indicators are registers (mainly the hospital discharge register and the population register) and health interview surveys. In addition, data for the Netherlands are available from national data sources for the majority of indicators (18 of 27) that are not available from international databases. These data are mainly based on the Dutch annual Quality of Life Survey (formerly POLS, now Health Interview Survey = Gezondheidsenquête) and GP (general practitioner) registries. However, for 23 (35%) out of the 66 indicators for which data are available breakdowns by socio-economic status and/or region are not available and for 21 (32%) of these 66 indicators the definition is not exactly the same as requested by ECHIM. See also the overview in Additional file 1 for more details on the availability per indicator. An example related to the possibility of breaking down by socio-economic status and region is “healthy life years”. Although these breakdowns are available for national health monitoring purposes based on the national HIS, Eurostat does not provide these dimensions for the European healthy life years indicator which is based on the European Union Statistics on Income and Living Conditions (EU-SILC). An example of a deviation from an ECHI indicator definition is cervical cancer screening. In the Netherlands women are asked whether they have undergone a cervical cancer screening test in the past 5 years instead of in the past 3 years because in the Dutch screening programme women aged 30–60 are offered a screening test once every five years. Additional file 3 lists the 21 indicators that do not match the ECHI indicator definition, along with a brief description of the difference.

Within the Joint Action for ECHIM we were not able to find, obtain or process adequate data for the following 9 (33%) out of the 27 indicators that are not available from international databases:

- self-reported prevalence of asthma;
- self-reported prevalence of COPD;
- self-reported incidence of injuries at home/leisure/school;
- self-reported incidence of road traffic injuries;
- self-reported fruit consumption;
- self-reported vegetable consumption;
- social support;
- participation in colon cancer screening;
- selected outpatient visits based on administrative sources.

For fruit and vegetable consumption, for example, no suitable data are available from the national HIS. The Food Consumption Panel study does contain detailed data on fruit and vegetable consumption, but these do not allow computation of estimates according to the ECHI definition.

### Data linkage possible in the Netherlands, but poor coverage hospital discharge registry

For the attack rate of acute myocardial infarction (AMI) and the attack rate of stroke only a combination of mortality data and hospital discharge records can provide a complete picture of the disease burden because a relatively large proportion of AMI and stroke patients die suddenly before reaching the hospital. To prevent double counting of events, these data should be linked at the patient level. For the ECHIM Pilot Data Collection, Statistics Netherlands computed the attack rates for AMI and stroke according to the ECHI definition, i.e. through linking hospital discharge and mortality data at the patient level. Because the hospital discharge data suffer from non-response from 2005 onwards, rates were calculated for 2004.

### Improvements over time: better estimates for low birth weight, practising physicians and practising nurses

Until 2001, Dutch data on low birth weight could only be based on a Health Interview Survey (POLS) that asks the parents about the birth weight of their children. Because health surveys are based on a sample of the total population, the number of respondents who are parents of newborn babies is relatively low. The estimates for low birth weight are therefore less robust than other health survey based estimates. Furthermore, recall bias will influence self-reported estimates. Therefore, a registry-based estimate is considered more appropriate for the indicator low birth weight. With the establishment of the database of the Netherlands Perinatal Registry (PRN-foundation) in 2001, such registry-based data have become available for the Netherlands and since 2009 Statistics Netherlands reports these registry-based data from PRN to the WHO-HfA database.

Regarding data on the numbers of physicians and nurses, until 2010 Dutch data were based on the register of (para)medical professions (BIG registry). In the BIG registry ‘physicians’ and ‘nurses’ refers to the concept ‘licensed to practise’. How many of them are actively practising and in which sector, is not known. ECHI (and Eurostat), however, requests ‘practising physicians’ and ‘practising nurses’ i.e. physicians and nurses that are ‘immediately serving patients’. Therefore, in 2010 Statistics Netherlands developed improved estimates on professionals working in the health care sector by combining the BIG registry with the SSB database (Sociaal Statistisch Bestand). This is a micro-integrated database of Statistics Netherlands with data from the municipal register, tax register, social security, and business register. This has resulted in estimates for the number of ‘professionally active physicians/nurses’ [15,16]. Statistics Netherlands calculated these estimates retrospectively for several years and delivered them to Eurostat, OECD and WHO. This is an improvement compared to the BIG registry based data, but does not yet fully meet ECHI requirements. Based on existing registries and databases it is, however, currently not possible to obtain estimates on physicians and nurses that are immediately serving patients.

## Discussion

Data availability in the Netherlands is good. However, for 21 out of the 66 indicators for which data are available the definition is not exactly the same as requested by ECHIM. This hampers the comparability of Dutch data with data for other countries and limits the possibilities for international benchmarking. Similarly, for 23 out of the 66 indicators for which data are available breakdowns by socio-economic status and/or region are not available. This hampers comparisons between regions and different socio-economic groups both within and between countries.

### Implementation of EHIS will improve data availability in the Netherlands

Although the data availability is already good in the Netherlands, implementation of EHIS will improve data availability further. The Netherlands did not participate in the first wave of EHIS (2006–2009). This first wave was carried out under a gentlemen agreement, which means that it was not mandatory for EU countries to conduct a national health survey following the EHIS guidelines [17]. The second wave of the survey (planned for 2013–2015) is currently being implemented under a Regulation. According to this implementing regulation the survey (meaning: the delivery of the variables specified by the regulation) is obligatory [18]. Therefore, all EU Member States will conduct EHIS in the second wave. In the Netherlands EHIS wave II has been integrated in the regular Dutch health interview survey between January and December 2014. After the implementation of EHIS wave II the data availability for the ECHI indicators will improve significantly for the Netherlands because EHIS is the preferred data source for about 20 ECHI shortlist indicators. Most EHIS questions for medical consumption, health status and lifestyle indicators are already included in the Dutch HIS. Some questions will be added in 2014 (i.e. the questions on fruit and vegetable consumption, on social support, colon cancer screening, and on the self-reported prevalence of asthma and of COPD) and some questions will be adapted to better match EHIS requirements. See also the detailed overview in Additional file 1 for the indicators included in EHIS.

For the EHIS questions on physical and sensory functional limitations, mental health, physical activity/exercise and alcohol consumption Statistics Netherlands has requested a derogation. These questions are necessary for the computation of some of the ECHI indicators for which the indicator definition was lacking or for which no preferred source was established yet at the start of the Joint Action (see Additional file 2). Reasons for the derogation relate to the wish to keep alignment with the regional Dutch HIS for some key questions, and the wish not to break long time trends in indicators used in national health monitoring. Such balancing between national and EU requirements is also common for other countries with a long history in national health reporting. Moreover, the uncertain legal basis for future EHIS waves after 2013–2015 makes it difficult for countries to decide on adapting national surveys to the EHIS questionnaire.

### Registries are a valuable source of information, but coverage of hospital discharge registries is point of concern

The detailed overview of data availability in Additional file 1 shows that registries such as GP registries and hospital discharge registries are a valuable source of health information in the Netherlands. For 6 indicators Dutch data were collected from GP registries within the ECHIM pilot data collection and/or within the pilot phase of the Eurostat diagnosis-specific morbidity statistics activities [14,19]. Sixteen countries (including the Netherlands) have carried out the Eurostat diagnosis-specific morbidity statistics pilot between 2007 and 2010. Eurostat activities on diagnosis-specific morbidity statistics are aimed at providing best national estimates based on administrative data and disease registers. The final aim is to set up a regular data collection on morbidity within the European Statistical System by 2020 [20].

For 8 indicators data are available from hospital discharge registries. Since 2005 the coverage of the voluntary Dutch hospital discharge register (Landelijke Medische Registratie, LMR; since 2013 called Landelijke Basisregistratie Ziekenhuiszorg, LBZ) has decreased because of the introduction of a new obligatory financial registration causing a lot of administrative burden for the hospitals [21]. The decrease in the coverage of the hospital discharge register threatens the data availability for most of the 8 ECHI indicators.

Because both HIS and registry-based data have advantages as well as disadvantages, ECHI proposed to use two indicator operationalisations for 8 indicators: one based on data from HIS and one based on national administrative or register data [3]. There are limitations to the comparability of national estimates based on administrative sources and registries, because health care systems and health information systems differ considerably between countries. On the contrary, EHIS-based estimates suit well the purpose of international comparison because a common methodology is underlying the gathering of EHIS data [3]. An important advantage of administrative sources and registers over surveys, however, is that they identify diseases based on medical diagnosis. However, several chronic diseases may remain undiagnosed, because people do not always seek medical help. Although EHIS-based estimates are likely to be influenced by sampling and reporting biases, it has the advantage that it is a population based survey and also includes patients that have not been in contact with the types of health care services that are covered in the register data. As EHIS is based on self-report, this advantage does not apply to diseases that require medical diagnosis such as diabetes and cancer. In addition, self-reporting does not allow describing diseases in terms of ICD (International Classification of Diseases) codes.

### Measured data from European Health Examination Survey are the preferred future source for blood pressure and body mass index

The detailed overview in Additional file 1 shows that currently health examination surveys (HES) are not an important data source for the ECHI indicators in the Netherlands. HES that include some form of physical examination, are complementary to HIS and medical registers because they can provide information on undiagnosed cases and measured data on determinants of major chronic diseases, which are not available from other data sources. Therefore, ECHIM has proposed switching to using the European Health Examination Survey (EHES) [22] as preferred data source for the indicators blood pressure and body mass index, once EHES will be fully implemented in a majority of EU Member States.

Between 2009–2011 twelve countries, including the Netherlands, piloted a HES in the working age population as part of the EHES Pilot Project. Within this framework the Dutch HES ‘Measuring the Netherlands’ (NL de Maat) was carried out in a national sample of over 4500 people (men and women) aged 18–70 years, which was in fact a full size HES. Although the initial low response rate necessitated a more intensified recruitment process, the results showed that a standardised European Health Examination Survey is feasible in the Netherlands [23].

The EU Member States are responsible for funding and conducting their national HESs, while the EU added value lies in promoting standardisation of national HESs. In the EU funded EHES pilot the EHES Reference Centre took care of the European level coordination, including the development of a European survey protocol. However, the future funding of the EHES Reference Centre is currently open and EHES is not a sustainable system yet.

### Data ownership in the Netherlands is scattered

The examples above illustrate that initiatives to improve harmonised data collection should be sustainable in order to safeguard future data collections and hence possibilities for monitoring trends. Strong national coordination for the regular collection of data in the area of public health and health care could contribute to sustainable collection of data. In Dare to Compare! it was, however, concluded that stronger national coordination and data ownership is needed in the Netherlands [9]. Also, Additional file 1 and the results of the Dutch pilot data collection during the Joint Action for ECHIM showed that data ownership in the Netherlands is scattered. Many different stakeholders, both public and private, collect (public) health data, without central steering. Moreover, not all data are freely available for research and monitoring purposes. This made it difficult to gather all necessary data [8].

The scattered data ownership situation in the Netherlands is a complicating factor in relation to the data coordination work for ECHI in particular and to the data coordination work for international data deliveries in general. More central coordination, including clear arrangements on the accessibility of publicly funded data sources for monitoring and research purposes, is therefore desirable. Since the beginning of the Joint Action for ECHIM, the Ministry of Health has been working on a plan to establish central coordination and steering for health data collections in the Netherlands. Progress has been made in bringing together municipal health services and Statistics Netherlands to jointly collect a set of HIS data [24]. Next, various Dutch organisations and institutes involved in lifestyle monitoring are now working together to improve lifestyle monitoring. This should prevent that multiple (different) national figures on the same indicator are published [25,26]. These activities are of relevance for the availability of Dutch ECHI indicator data.

## Conclusions

1. Data availability in the Netherlands is good. For all of the 48 ECHI indicators that can be extracted from international databases, data for the Netherlands are available. Moreover, the Netherlands can provide data from national data sources for the majority (18 of 27) of ECHI indicators not available from international databases. Breakdowns by socio-economic status and region are not possible for 23 (35%) of the total of 66 indicators for which data are available and for 21 (32%) of these 66 indicators the definition is not exactly the same as requested by ECHIM.
2. Since 2009 several improvements in harmonised data collection have been achieved, which have already contributed or will significantly contribute to improvements in the data situation for the ECHI indicators in the Netherlands, as well as for other EU countries. Examples are the calculation of better estimates for several indicators at national level and EU-wide developments in EHIS, EHES and the Eurostat diagnosis-specific morbidity statistics activities. Such health surveys and other data collections should become sustainable data sources in order to provide possibilities for monitoring trends. At the moment, however, the sustainability of these developments is not yet guaranteed.

## Additional file

Additional file 1:
**Detailed overview 75 ECHI indicators.xls.** Detailed overview of data availability between 2011 and 2013 for the 75 indicators for which ECHI indicator definitions are finished and for which the Joint Action for ECHIM made a decision on the preferred data source. Additional file 2:
**21 ECHI indicators unfinished at start JA for ECHIM.xls.** The 21 ECHI indicators for which the indicator definition was lacking or for which no preferred data source was established yet at the start of the Joint Action for ECHIM. Additional file 3:
**21 ECHI indicators with definition problems.xls.** Description of the difference from the ECHI indicator definition for the 21 indicators that do not match the ECHI indicator definition.

## Abbreviations

AMI: acute myocardial infarction
BIG: Dutch register of (para)medical professions (Beroepen in de Individuele Gezondheidszorg)
COPD: Chronic Obstructive Pulmonary Disease
ECHI: European Community Health Indicators
ECHIM: European Community Health Indicators Monitoring
EHES: European Health Examination Survey
EHIS: European Health Interview Survey
EU: European Union
EU-SILC: European Union Statistics on Income and Living Conditions
GP: General Practitioner
HES: Health Examination Survey
HIS: Health Interview Survey
ICD: International Classification of Diseases
LBZ: Landelijke Basisregistratie Ziekenhuiszorg
LMR: Landelijke Medische Registratie
OECD: Organisation of Economic Co-operation and Development
POLS: Dutch annual Quality of Life Survey
PRN: Netherlands Perinatal Registry
RIVM: National Institute for Public Health and the Environment
SSB: Social Statistical Database (Sociaal Statistisch Bestand)
WHO: World Health Organization
WHO-HfA: World Health Organization Health for All database
